# Routine dipstick urinalysis in daily practice of Belgian occupational physicians

**DOI:** 10.1186/0778-7367-70-15

**Published:** 2012-06-21

**Authors:** Lutgart Braeckman, Eva Haak, Lieve Peremans

**Affiliations:** 1 Department of Public Health, Ghent University, De Pintelaan 185, 9000, Ghent, Belgium; 2 Provikmo, Occupational Health Service, Dirk Martensstraat 26/1, 8200, Bruges, Belgium; 3 Department of Primary and Interdisciplinary Care, University of Antwerp, Universiteitsplein 1, 2610, Wilrijk, Belgium

**Keywords:** Evidence-based practice, Occupational health, Guidelines, Health surveillance

## Abstract

**Background:**

Little work has been done to assess the quality of health care and the use of evidence-based methods by occupational physicians in Belgium. Therefore, the main objective is to describe one aspect of occupational health assessments, namely the common use of dipstick urinalysis, and to compare the current practice with international guidelines.

**Methods:**

A self-administered questionnaire was mailed to 211 members of the Scientific Association of Occupational Medicine in the Dutch speaking part of Belgium.

**Results:**

A total of 120 occupational physicians responded, giving a response rate of 57%. Dipstick urinalysis was a routine investigation for the vast majority of physicians (69%). All test strips screened for protein and in 90% also for blood. Occupational health services offered clinical tests to satisfy customer wants as international guidelines do not recommend screening for haematuria and proteinuria in asymptomatic adults. A lack of knowledge concerning positive testing and referral criteria was demonstrated in almost half of the study participants.

**Conclusions:**

Belgian occupational physicians still routinely perform dipstick testing although there is no evidence to support this screening in healthy workers. To practice evidence-based medicine, occupational physicians need more instruction and training. Development and implementation of more guidelines is not only of use for the individual practitioner, it may also enhance professionalization and efficiency of occupational health care.

## Background

In recent decades, evidence-based methods have been successfully applied in many areas of health care and prevention [1,2]. However, the development and appropriate use of evidence-based guidelines seem more problematic in the field of occupational medicine [1,3]. Research shows that doctors’ attitudes towards guidelines are a good predictor of their intention to use them [3]. Only a few studies have assessed the attitudes and perceptions of occupational physicians (OPs) towards evidence-based medicine (EBM) and the majority appears to have a positive attitude [3-7]. The average degree of adherence to recommendations in guidelines is no higher than 60 to 70%, but there is a large variation between physicians and between different guidelines [8]. Lack of time, limited availability of relevant guidelines and poor EBM skills are the main barriers that prevent occupational physicians from practicing evidence-based medicine [3-7]. In addition, some authors argue that the application of evidence-based methods is hampered because occupational medicine is practiced within a framework of labour law and governmental regulations [7,9].

In Belgium, occupational health care is compulsory and the current legislation determines to a considerable extent its context and content [10]. All workers (3.7 millions), except the self-employed (0.7 million), benefit from comprehensive occupational health support. About half of the workforce undergoes an annual health examination and another fifth has an assessment every 3 or 5 year. The content of the periodical health surveillance including the use of specific clinical tests is based on some legal requirements and is not underpinned by any sound evidence. Occupational health is completely financed by the employers and depends largely on the total number of health assessments. Like in many other countries, there are too few occupational physicians and the active ones are under pressure to perform all the clinical examinations instead of providing hazard definition and measurements, risk management, health and safety information and training. The number of physicians working in Belgian occupational health services (OHS) is estimated at 1100 (800 full-time equivalents), giving 30 (22 FT) OPs per 100.000 workers or an understaffing of 30%. There are no readily obtainable comparable data, but Nicholson presented a crude guide to the diversity of access to OPs within Europe, ranging from 5 physicians per 100.000 in the UK to 61 physicians per 100.000 workers in Finland [11]. In this context, evidence-based occupational medicine is not considered a priority and Belgium is lagging behind in the development and implementation of EBM in the occupational health setting. While the Netherlands and the United Kingdom have published several evidence-based and clinical practice guidelines, only recently a first recommendation, concerning job-related fitness tests for firemen, was developed by a Belgian expert panel [12-14].

To obtain some insight to what extent Belgian OPs integrate evidence in their medical decisions during consultation, we gathered data and information on one routine activity in occupational health surveillance, in particular urinalysis. Although the screening of all workers at pre-employment and annual health examinations for albumin and glucose in urine is no longer a legal requirement (Publication in 2003 of the Belgian Royal Decree on health surveillance of workers), dipstick testing is still common in daily practice. Dipstick urinalysis is simple and quick to perform, has no morbidity (other than sometimes labelling a healthy person as sick), and is among the most commonly performed screening tests [15,16]. However, whether physicians should routinely screen for haematuria and proteinuria in asymptomatic persons is a point at issue [15-18]. The most common causes for haematuria in adults include urinary tract infections, urolithiasis and urological malignancy. For the detection of haematuria, the specifity of a heme dipstick positive at 1+ or greater has been reported as 65.0% to 99.3% when microscopic haematuria is used as the golden standard. Heavy exercise or prolonged recumbency can produce haematuria in normal adults. The predictive value is low (0% to 2% for significant disease) and once haematuria is confirmed, its cause should be investigated through a comprehensive history, a focused physical examination, laboratory studies, an image-based assessment of the upper urinary tract and a cystoscopic evaluation of the lower urinary system [15-18]. Clinicians are concerned about whether an evaluation for micro haematuria is justified considering the associated risks: unnecessary exposure to radiation, potential allergic reactions to the dyes used in intravenous pyelograms and complications from renal biopsies [15-17]. Furthermore, the cost of screening and a complete urinalysis evaluation for haematuria are substantial [15,16,19]. An estimated 150 million dollars is spent on dipstick urinalysis annually in the United States [15]. The physician should be guided by the patient’s history and physical examination whether testing for haematuria is appropriate. Risk factors for significant underlying disease include: age > 40 years, tobacco use, analgesic abuse, history of pelvic irradiation and exposure to occupational toxins such as dyes, benzenes and aromatic amines [15-17].

Assessment of albumin and/or protein excretion in the urine is a key step in the early detection and appropriate management of chronic kidney disease (CKD) [20-22]. However, the approach to testing for albuminuria/proteinuria in the community is variable, often suboptimal and hampered by a paucity of high level clinical evidence to guide who should be screened, when and how often they should be screened and what test should be employed. Dipstick testing with protein or albumin reagent strips has been long-established in routine clinical practice but its usefulness as a screening strategy is significantly limited by poor sensitivity and specificity. For screen-positive patients (dipstick protein ≥ 1+), testing should be repeated since transient albuminuria/proteinuria is commonly seen following febrile or other acute medical illnesses (e.g. urinary tract infection). If two or more albumin/protein measurements are elevated, referral to a nephrologist is needed to investigate kidney function and diagnose kidney damage. Comparable with a urological evaluation, the health risks and economic costs of the establishment of a nephrological abnormality (urine sediment, imaging tests, kidney biopsy,…) can be substantial. At present, population-based screening is not recommended, but targeted screening should be performed for all patients who are at high risk of kidney disease (patients with hypertension, diabetes, cardiovascular disease). Over the last years, progress has been made towards developing a global position statement and it is likely that measuring albumin to creatinine ratio (ACR), preferably on a first void morning specimen, will increasingly be used for CKD screening in all at-risk individuals [20-22].

In this paper, the current Belgian practice to include dipstick urinalysis in occupational health assessments is described and compared with scientific evidence and international recommendations. In order to alter inappropriate screening policy and to provide better services, we need to understand why OPs still perform dipstick testing.

## Methods

To fulfil the objectives of our study, a questionnaire was developed on the basis of a comprehensive literature review of evidence-based occupational medicine and existing guidelines on urinalysis [15,17,18,20,21]. The questionnaire was pilot-tested in a group of seven occupational physicians and modified based on the feedback we received from this group. More response categories were made available for some questions and two additional questions concerning the cut-off point for referral to an urologist/nephrologist were included. The revised questionnaire consisted of five parts [1] physicians’ gender, employment and years of experience in occupational medicine; [2] urine collection, used methods and screening tests; [3] who is screened; [4] use of a protocol or recommendations; [5] referral criteria and management of positive test results.

Because the questionnaire was only available in Dutch, the study population of this survey consisted of the members (some of whom were in training) of the Flemish Scientific Association of Occupational Medicine (VWVA). These are all Dutch-speaking physicians, employed at both external and internal OHS across the northern part of Belgium. Approval was obtained from the board before physicians were contacted by e-mail. The questionnaire was administered online to 211 occupational physicians during March 2010. The survey was anonymous and the whole population received one reminder after three weeks. As the study was descriptive in nature, no statistical analyses were carried out on the data.

## Results

In total, 120 physicians (57%) responded to the questionnaire. Table 1 gives an overview of the descriptive results. Of the 120 respondents, 52% (n = 62) were men, 83% (n = 100) were employed at Belgian external OHS and 17% (n = 20) at internal OHS (some large companies have developed their own in-house health care services). For the whole sample, the mean years of experience as an occupational physician was 13 (range 1 to 35).

**Table 1:** Descriptive results of the study variables (n = 120)

**Variables**	**n**	**%**	**Internal OHS (n)**	**External OHS (n)**
	**120**	**100**	**20**	**100**
**Gender**				
Men	62	52%	15	47
Women	58	48%	5	53
**Who reads the tests**^**a**^				
Nurse	110	85%	17	93
Doctor	6	5%	3	3
Automatic device	6	5%	0	6
Nurse and automatic device	7	5%	0	7
**Screening for haematuria**				
Yes	108	90%	16	92
No	12	10%	4	8
**Who is screened**				
All workers	83	69%	19	64
All except low risk	37	31%	1	36
Those at-risk	0	0%	0	0
**Presence of protocol**				
Yes	100	83%	18	82
No	18	15%	2	16
No idea	2	2%	0	2
**Positive haematuria**^**b**^				
Correct answer	55	48%	10	45
Not correct	59	52%	8	51
**Positive proteinuria**^**c**^				
Correct answer	55	46%	8	47
Not correct	65	54%	12	53
**Referral after positive test**				
Yes	69	57%	7	62
No	12	10%	5	7
If presence of risk factors	39	33%	8	31

^a^ more than one answer possible.

^b^ n = 114 : 6 physicians did not test for heme; dipstick positive at equal or greater than 1+.

^c^ dipstick positive at equal or greater than 1+.

Regarding the practical aspects of the urine collection, subjects were requested to provide a urine specimen in a disposable container at the beginning of the medical examination. Test strips were read within a few minutes after voiding by the nurse in 85% of the samples, by the physician in 5%, an automatic analysing system was used in 10% and in another 5% it was done both by the nurse and a device (more than one answer possible).

The majority of the OPs (69%) confirmed that dipstick urinalysis was part of the routine investigation for the majority of workers under occupational health surveillance. Visual Display Unit operators and apprentices were not screened, usually because of time shortage. None of the physicians indicated a strategy of solely screening workers with a high-risk profile (e.g. increased risk caused by occupational exposure to chemicals). Standard dipstick tests always included screening for protein and glucose while additional testing for haematuria was done in 90%.

According to 85% of the physicians, the group of employees screened was imposed by procedures of the occupational health service.

A dipstick test result is considered positive at a colour indication of ≥ 1+ for blood and/or protein. Only 48% of the physicians gave the correct answer for haematuria and 46% for proteinuria. In the case of a positive reading for blood, 91% of the physicians checked for false positive causes (e.g. strenuous exercise), 87% asked for symptoms of urinary tract infections and 22% requested a further urine sample. When a test result was positive for proteinuria, 50% asked for high-risk factors for chronic kidney disease. To exclude transient causes of proteinuria, 15 physicians repeated the test after 2 weeks.

Persons with dipstick-positive haematuria and proteinuria were promptly referred by 66 physicians. According to 36% (n = 43) of the respondents, additional risk factors (e.g. older age) were needed to send workers for further assessment.

## Discussion

This descriptive study sought to evaluate current practice in occupational health surveillance, the focus being centred on urinalysis. Our results document the informal knowledge that dipstick urinalysis is being conducted in the vast majority of health examinations, irrespective of the workers’ risk profile.

To our knowledge, this is one of the few papers exploring the quality of occupational care in Belgium and this is the most important strength of the study. However, there are a number of limitations that have to be mentioned. First, a measurement bias cannot be ruled out. The instrument used in this study was a self-developed questionnaire and its validity and reliability were not investigated. A second weakness is that only the members of the Flemish Scientific Association of Occupational Medicine participated in this survey. This organisation represents one third of all OPs in Belgium and although a relatively large number of OPs (n =120) did respond, selection bias may have been introduced. It is possible that non-members have a different opinion and that OPs who do not routinely perform urinalysis, considered this questionnaire irrelevant and did not complete the survey. Nevertheless, respondents were employed at both types of OHS, representing 11 from the 14 certified external OHS (all the large OHS were included) and 20 in-house OHS. OHS are accredited and they usually have a certification for providing services to all workers as well in Flanders as in Wallonia. Therefore, we feel confident that the present results are representative for the whole group of active OPs.

A possible explanation for the usual practice in relation to urinalysis may be the lack of knowledge about the appropriate use and significance of tests among health care professionals. In many countries, numbers of diagnostic tests are growing and attempts have been made to change test ordering performance effectively and bring it into line with existing evidence or guidelines on optimal testing [23]. Although there is surprising variation among international guidelines, no major organization currently recommends screening healthy asymptomatic adults for haematuria and proteinuria. Belgian OPs do not have the choice; they are required by the procedures of their OHS to perform dipstick testing. From our own experience and former discussions with employers and employees, we know that clients more actively ask for tests. They expect a minimum of health surveillance tests such as anthropometric measurements, blood pressure reading, urinalysis and no longer performing these clinical tests could be considered as providing inadequate services and a try to take away acquired rights. In this manner, OHS may be afraid to lose business and they adopt a commercial approach by keeping unnecessary tests in the package they offer to their clients.

Besides the reduced physicians’ autonomy, some other issues must be considered; only half of the participants formulated the correct answer on the cut-off point for referral (dipstick positive at the level of +1) or follow-up. We did not ask for underlying reasons for this poor performance but previous surveys among OPs revealed that most of them consult the most up-to-date evidence in their field infrequently and that they make only sporadic use of practice guidelines. In addition, they possess limited skills in evidence-based medicine and the time required to keep up with the scientific evidence available is lacking [3,4,6,7]. These factors influence subsequently the quality of health care and advice they give to their clients. Instruction and training seems to be needed for most occupational physicians to increase their searching and critical-appraisal skills [3,9]. A study by Hugenholtz et al. demonstrated that an intervention with multi-faceted evidence-based medicine was a useful method to enhance professional performance [5]. However, the intervention was very time consuming. Since time limitations form a major problem, it would be helpful to quantify the amount of time OPs spend per clinical consultation and the proportion of time they spent on clinical activity compared to non-clinical activity [3,4,6,18]. As suggested by Adeodu et al., these results may help in developing and enforcing better standards for allocating occupational physicians’ time between clinical and non-clinical activities such as training [3].

In addition, further evidence-based guidelines in occupational health should be developed and implemented, as they are one of the most promising and effective tools for improving the quality of health care [1,8]. The development of such recommendations or guidelines is nevertheless labour intensive and expensive. Another problem is that such guidelines do not implement themselves. Therefore, additional tools like employer or employee versions of the guideline, checklists, indicators, and audit instruments should equally be developed by guideline developers, preferably in collaboration with different stakeholders.

In conclusion, the present study shows that occupational physicians tend to depend on their routines which are not always in line with evidence-based guidelines. To enhance professional performance, more efforts should be made to support occupational physicians in the uptake and maintaining of knowledge. Furthermore, to improve the quality of occupational health care, occupational health institutions, medical universities and scientific societies should be stimulated to take more initiatives to develop and implement evidence-based guidelines relevant to occupational practice.

## Competing interests

The authors declare that they have no competing interests.

## Authors’ contributions

LB coordinated the project and drafted the manuscript. EH was responsible for the study protocol and the field work. LP helped with the review of the literature and the design of the study. Moreover, EH and LP evaluated the results and commented on the manuscript. All authors have read and approved the manuscript as submitted.
